# Multivariate Thermo-Hygrometric Characterisation of the Archaeological Site of Plaza de l’Almoina (Valencia, Spain) for Preventive Conservation

**DOI:** 10.3390/s130809729

**Published:** 2013-07-29

**Authors:** Ángel Fernández-Navajas, Paloma Merello, Pedro Beltrán, Fernando-Juan García-Diego

**Affiliations:** 1 Departamento de Física Aplicada (U.D. Industriales), Universitat Politècnica de València, Av. de los Naranjos s/n, Valencia 46022, Spain; E-Mails: afnavajas@fis.upv.es (Á.F.-N.); palomamerello@outlook.com (P.M.); pbeltran@fis.upv.es (P.B.); 2 Instituto Valenciano de Conservación y Restauración de Bienes Culturales (IVC+R), Complejo Socio-Educativo de Penyeta Roja s/n, Castellón 12080, Spain; 3 Centro de Tecnologías Físicas, Unidad Asociada ICMM- CSIC/UPV, Universitat Politècnica de València, Av. de los Naranjos s/n, Valencia 46022, Spain

**Keywords:** microclimate monitoring, archaeological preservation, temperature and relative humidity sensors

## Abstract

Preventive conservation requires monitoring and control of the parameters involved in the deterioration process, mainly temperature and relative humidity. It is important to characterise an archaeological site prior to carrying out comparative studies in the future for preventive conservation, either by regular studies to verify whether the conditions are constant, or occasional ones when the boundary conditions are altered. There are numerous covered archaeological sites, but few preventive conservation works that give special attention to the type of cover installed. In particular, there is no background of microclimatic studies in sites that are in the ground and, as in the Plaza de l’Almoina (Valencia, Spain), are buried and partially covered by a transparent roof. A large effect of the transparent cover was found by the sensors located below this area, with substantial increases in temperature and a decrease in the relative humidity during the day. Surrounding zones also have values above the recommended temperature values. On the other hand, the influence of a buried water drainage line near the site is notable, causing an increase in relative humidity levels in the surrounding areas. Multivariate statistical analyses enabled us to characterise the microclimate of the archaeological site, allowing future testing to determine whether the conservation conditions have been altered.

## 1. Introduction

Preventive conservation of archaeological sites is understood as the whole control process of the deterioration factors in order to prevent damage to the cultural heritage before it occurs and minimise future interventions [1].

The conservation of an archaeological site is particularly influenced by the thermo‐hygrometric features of the environment in which it is located, which may lead to material disintegrations and biological problems, *etc.* Thus, monitoring and control of the physical parameters of temperature and relative humidity become a priority [2].

It is important to characterise an archaeological site with a view to carrying out comparative studies in the future for its preventive conservation, either regularly, in order to verify whether the conditions are constant, or occasionally, when the boundary conditions are altered. It is also important after any change in the environment to first ascertain if the resulting microclimate is suitable according to the experience of other researchers and standards, and, secondly, if deterioration in the site occurs, to know the microclimate that has led to this phenomenon.

The control of these parameters has been studied in churches [3–5] and museums [6–9]. Microclimatic monitoring studies have also been conducted in open archaeological sites [10,11]. In the case of the ruins of Ariadne’s house in Pompeii [11], as a consequence of this study it was possible to propose corrective measures on the covertures that helped improve preventive conservation of the site and its frescoes.

In the case of closed or buried archaeological sites (hypogeum) under climate control systems, it is very important to control the operation of the latter, as well as to prevent harmful combinations of temperature and relative humidity that may lead to the appearance of fungi (high temperature and humidity) or drying of the substrate. A monitoring study of temperature and relative humidity (RH) was carried out at the hypogeum archaeological site of Carcer Tullianum (Rome, Italy) [12]; in that work, statistical tools for exploratory purposes, such as box and whisker plots, were used, but statistical comparative techniques were not applied, which is one of the aims of this paper.

There are many examples of covered and visited archaeological sites that can be found, as well as the archaeological crypt in Paris (France), the ruins of the History Museum of Barcelona (Spain), the Saint Laurent church and Saint Oyand crypt (Archaeological Museum of Grenoble, France), and the archaeological site of Saint-Pierre Cathedral in Geneva or the Carcer Tullianum [12].

The authors of [13] discuss the installation of different types of coverage on archaeological remains. Thus, covertures with different designs and structures are installed, but which could be included in two general classes: opaque or transparent covers. Opaque covers are more effective, but generally hamper the visitor's view, so there are many cases in which it was decided to install a transparent cover. However, it is in the case of transparent covers where the control of thermo‐hygrometric parameters is most needed [11].

The l'Almoina Archaeological Centre (Valencia, Spain) comprises an area of about 2,500 m^2^. It houses the archaeological excavations performed between 1985 and 2005 in the city of Valencia [14]. This work has led to the discovery of several monumental buildings, inscriptions, loose architectural elements, more than 1,000 coins and 500 exhibition quality ceramic pieces [15], and has also given rise to a vast body of technical documentation.

The most interesting facet of this site is the buildings, a continuous overlapping of constructs, forming a complete and well-preserved compendium of history and urban development of the city of Valencia from its founding to the present. Among them, we find the Islamic Alcazar [16], the first city (the Republican) represented by the thermals [17], the remains of the Roman Empire (the forum and the Curia) [18,19], and martyrdom and Episcopal area from the Vizigothic stage [19,20].

**Figure 1.**
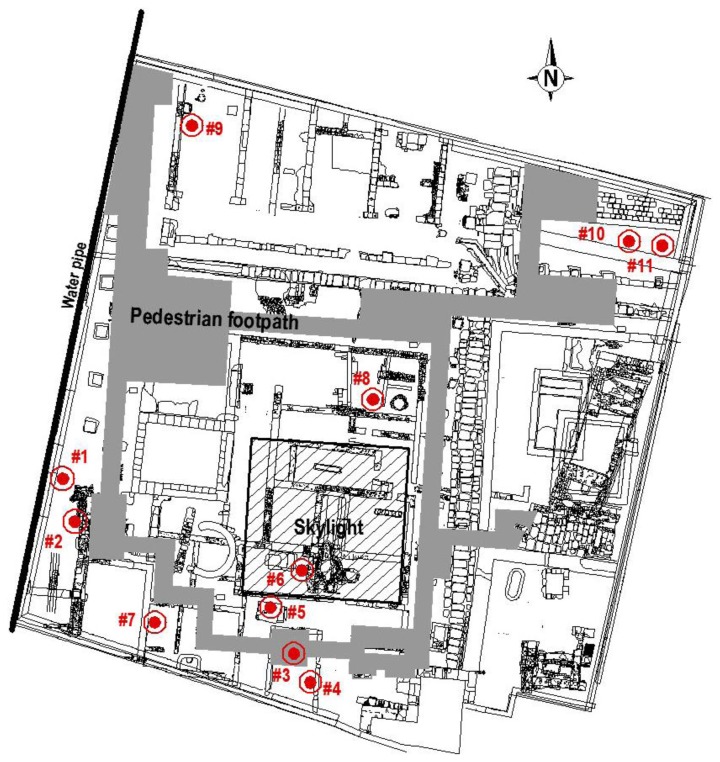
Plan of the archaeological site and location of sensors.

An intervention was conducted in the years 2005–2007 for the development and construction of the Archaeological Centre. To protect the ruins, a concrete structure adapted to the unique archaeological site was built. In addition, as shown in Figure 1, a glass cover (25 × 25 m) was installed, which allowed passers-by a glimpse of the archaeological remains.

This work deals with the multivariate analysis of microclimatic conditions at the archaeological site of the Plaza de l'Almoina, which allows us to characterise the site with the aim of carrying out comparative studies in the future for preventive conservation of this site when implementing changes in the climate control system or in the architectural design. To this end, we shall perform different statistical analyses, not frequently used in cultural heritage, such as mean daily trajectories, bivariate plots and cluster analysis, with the aim of illustrating mainly restorers and conservators of archaeological sites how to proceed when monitoring systems are affected by technical and economic limitations, resulting impossible to install an ideal sensor network.

## 2. Materials and Methods

### 2.1. Description of Data Loggers

Each RH data logger (Datalog Hygrochron DS1923) contains a humidity sensor with an accuracy of ±5% [21]. Although this model can also record temperatures, it was decided to use independent devices (Datalog Thermochron DS1922L) for the temperature monitoring [22], which has the same accuracy (±0.5 °C) as the DS1923. The reason was to expand the data storage capacity of the monitoring system. A set of 22 data loggers, 11 of each model, were purchased directly from the manufacturer (Maxim Integrated Products, Inc., Sunnyvale, CA, USA) and calibrated prior to their installation, as described below. These devices resemble button like batteries, with 17.4 mm of diameter and 5.9 mm of height. Each pair of DS1923 and DS1922L data loggers was placed close to each other.

### 2.2. Installation of Data Loggers

The monitoring study started on 22 February 2010 and ended on 5 July 2010, when 11 probes were installed in the archaeological site of Plaza de l’Almoina (location shown in Figure 1). The monitoring period was selected as it provided a representation of cold and warm season, especially considering that the summer is the most interesting period because it presents the most unfavourable microclimatic conditions for conservation given the location of the archaeological site and the transparent cover installed.

**Figure 2.**
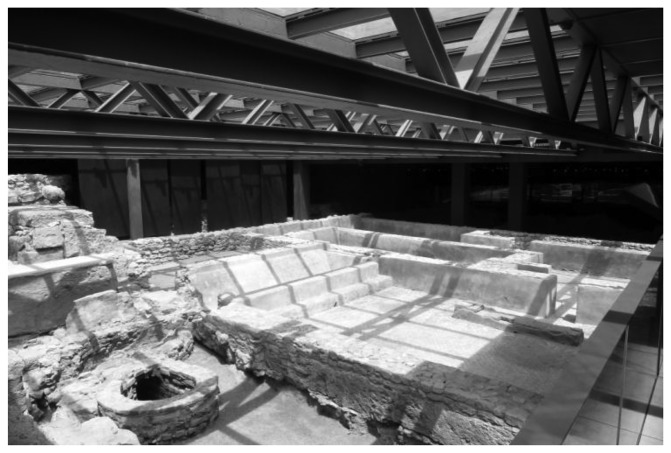
Light coming from the skylight, which affects only part of the archaeological site.

**Figure 3.**
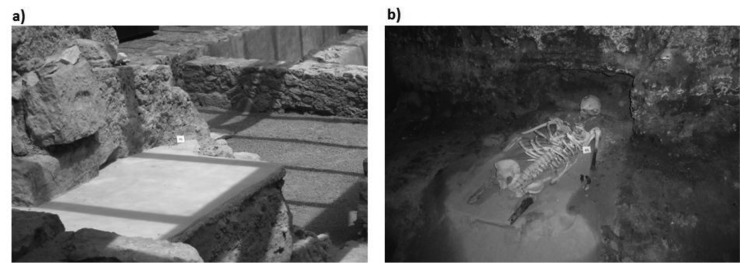
Location of sensors with singular trajectories. **(a)** Location of sensor #6, just below the skylight; **(b)** Location of sensor #1, next to skeletal remains (the water pipe is located just above).

Meteorological data on temperature and RH provided by the Environment Department of the Universitat Politècnica de València are also available, and will serve as an outdoor reference. In order to analyse the effect that light passing through the skylight (Figure 2) has on the archaeological site, sensor #6 was installed immediately below it (Figure 3a).

All probes were placed on the ground (Figure 3), except probe #2, which was placed in a recess in the archaeological stone wall. Note that at the west of the archaeological site a boundary water pipe from the 20 s was located (Figure 1), not canalised with PVC, so sensor #1 was located just near the wall facing the pipe (Figure 3b).

### 2.3. Calibration of Sensors

Calibration of the sensors was performed with two calibration experiments separated in time, in order to study if measurements from one or more sensors were biased compared to the average recorded by all sensors. Thus, the average sensor bias was corrected for all data. The calibration procedure and the results of the bias can be found in [11] because the sensors were the same.

### 2.4. Frequency of Data Recording

The monitoring study began on 22 February 2010 and ended on 5 July 2010, resulting in a total period of 133 days. All data loggers were programmed to register one measurement every 30 min, which implies 1,440 recorded values per month (i.e., 30 days × 24 h/day × 2 data/h).

### 2.5. Statistical Analyses

#### 2.5.1. Normal Probability Plot

Normal distributions are extremely important in statistics, and are often used in the natural and social sciences for real-valued random variables whose distributions are unknown [23]. The normal probability plot is a graphical technique for normality testing, assessing whether or not a data set is approximately normally distributed. The data is plotted against a theoretical normal distribution, so that the points should approximately follow a straight line, and those departures from this line indicate departures from normality. This is a quantitative technique suitable for researchers trained in statistical analysis.

In our case, we are interested in detecting those sensors whose differences from the average are abnormal. We work on this paper with two different averages. First, we work with the average of all sensors (calculated considering sensors from #OUT to #11). Second, since we are interested in detecting differences within the archaeological site, we work with the average of inner sensors (calculated considering sensors from #1 to #11).

#### 2.5.2. Daily Mean Trajectories

Mean daily trajectories are used in several fields of science, but rarely applied in microclimate monitoring of cultural heritage [11]. Mean daily trajectories are calculated as the average of the data from each sensor per fraction of time (in this case, every hour) for the entire date range of interest. This plot summarises the information of the selected time period, avoiding excessively large plots and stationary periods and allowing simple comparison of different sensors. This technique enables us, for example, to detect anomalous data quickly and effectively, such as time bands where direct sunlight affects the paintings, causing a rise in temperature.

#### 2.5.3. Cluster Analysis

Cluster analysis is the name of a group of multivariate techniques whose primary purpose is to gather objects based on their characteristics, attempting to maximise the homogeneity of objects within clusters while simultaneously maximising the heterogeneity between clusters [24].

In this study, the aim of using cluster analysis was to define a taxonomy of sensors, to characterise the different zones of the archaeological site from their average and daily variability in RH and temperature.

In this paper, the squared Euclidean distance is used as a measure of similarity between observations and hierarchical method of k-means is applied. Note that analyses were also performed for the normalised data (both variables having equal weight in the analysis); as the obtained results were identical, it was decided to present the results for the original variables because of their physical interpretation. All cluster analyses were performed using the software SPSS 16 [25].

#### 2.5.4. Contour Plots

The purpose of including this type of graph in the article is twofold: on one hand they are useful as preliminary study to give an overview of the microclimatic situation of the site; on the other hand, they serve to discuss how the proposed techniques improve the results obtained from contour plots.

These plots were made with a CAD program, connecting each sensor to the closest one with a straight line, forming a triangle. Each line is graded according to the initial sensor value and the final one, resulting in a number of marks where each represents a value of the physical parameter. Linking the marks of the same value by splines, you get a line plot representing an approximation of the gradient of the physical parameter.

## 3. Results and Discussion

### 3.1. Classical Data Analysis

#### 3.1.1. Descriptive Data Analysis of Mean Trajectories

Knowledge about ideal or limit values of microclimate parameters for conservation of cultural heritage is still poor [12]. The Italian UNI 10829 [26] and DM 10/2001 [2] are currently the approved standards on this matter. According to [2,26], the recommended range of RH and temperature for stones and rocks is 40–60% and 19–24 °C, and a maximum daily variation of 6% in RH is recommended, although there is no available data for temperature.

**Figure 4.**
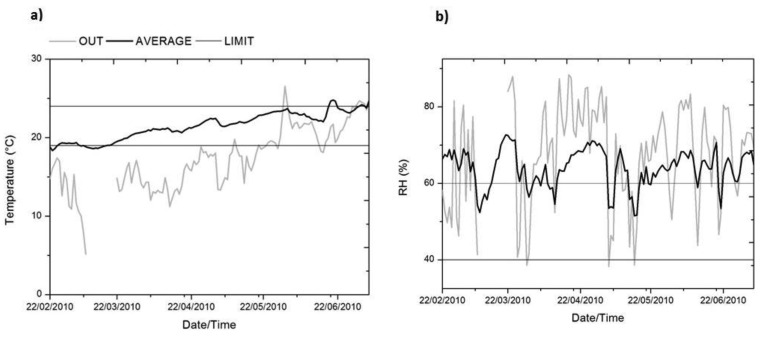
Daily averaged data for the whole monitoring period (48 data/day), for the outdoor sensor and the average of interior sensors (11 data/day). The value 0 on the horizontal axis coincides with the date 22 February 2010. **(a)** Temperature; **(b)** relative humidity (RH).

Figure 4a allows us to detect the growing trend for the temperature data in the monitoring period, as expected. Moreover, Figure 4 verifies how, *a priori*, the architectural design of the archaeological site mitigates the high variability of the Mediterranean climate of the city of Valencia (Spain), in temperature and especially in RH.

#### 3.1.2. Contour Plots of Temperature, RH and Water Vapour Pressure

To produce these contour plots, data from sensor #2 were removed from the graphs, as it was installed in a recess of the wall without contact with soil moisture, resulting in a distortion of the plots.

**Figure 5.**
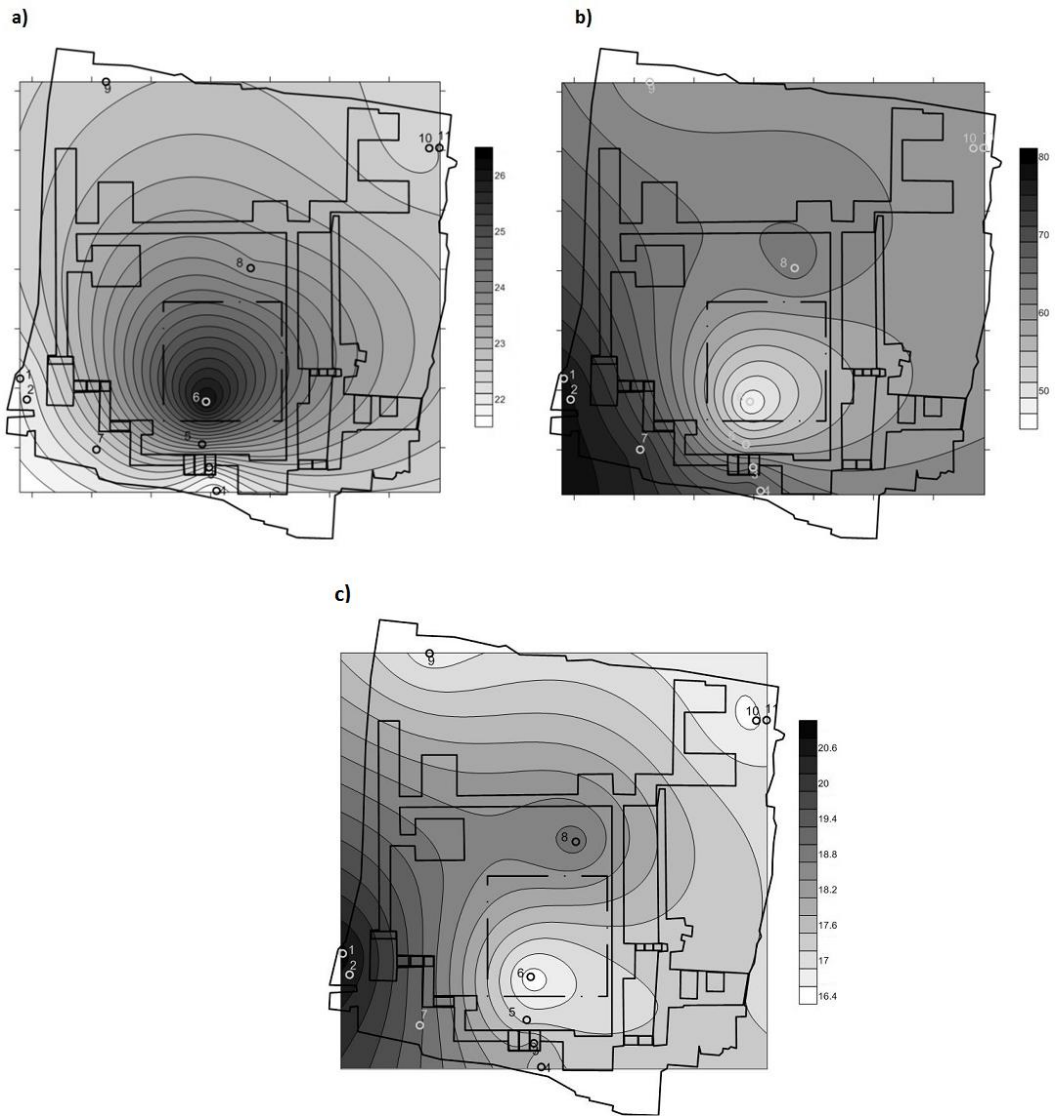
Contour plots, averaged data from May to July 2010 from 00:00 to 23:59 h (3,120 data/sensor), **(a)** temperature (°C); **(b)** relative humidity (RH, %); **(c)** water vapour pressure (mbar).

Figure 5a shows how temperature is influenced by the effect of the glass cover, which caused an anomalous performance of sensor #6, placed just below the skylight. In the case of RH, Figure 5b, the influence of the transparent cover results in a rise in temperature and consequently a decrease in RH. As Figure 5b shows the remaining areas are influenced by the RH gradient, which decreases as we move away from the water drainage line as an effect of the movement of the water as indicates the water vapour pressure (Figure 5c). Note that, if there is a difference pressure in a closed site, as pressure tends to equalize, thus there will be movement of the water content.

Temperature graphs and RH gradients are widely used in cultural heritage [1,27], but are limited because they rely on the concept of representing infinite points from a finite number of sensors. In our case, it is clear that under the skylight, where there is only one sensor (#6), the graph is distorted and centred on this sensor.

**Figure 6.**
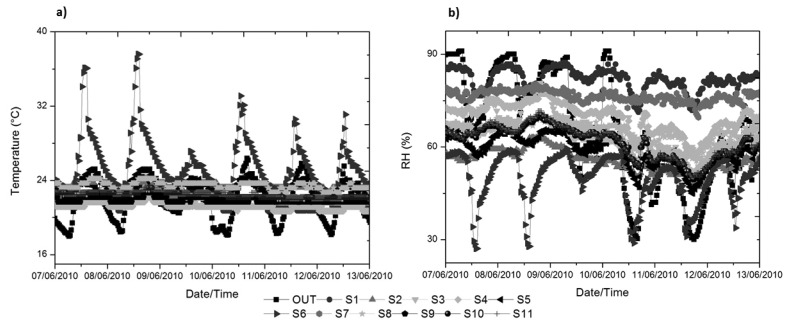
Time series of all sensors, for the week from 7 July 2010 to 13 July 2010, **(a)** temperature; **(b)** relative humidity (RH).

To avoid this problem, in the following sections a more comprehensive and quantitative methodology is applied to characterise the archaeological site. These techniques surmount the inherent difficulties of interpretation of a large number of sensors as shown in Figure 6, where it is very difficult to draw useful conclusions.

### 3.2. Multivariate Methodology Proposed

#### 3.2.1. Detecting Singular Trajectories (Normal Probability Plot)

In this paper, we shall understand as outliers those values far from other values, whether close to the centre of the distribution or not, which can be ruled out because they distort the conclusions. In the case of time series of physical parameters such as temperature and RH, we define as outliers those incorrect records that exceed the acceptable values for a physical parameter trajectory. In our case, the software used [28] highlights those records that exceed a maximum variation every half hour defined by the user; in this case, it was fixed in a range of ±8 °C in temperature and ±20% RH. In this case, no outliers which could be eliminated were identified.

However, sensors with behaviours unusual or distinct from the rest may appear, for which the information is of great interest to understand the reality of the archaeological site.

To detect the sensors with singular trajectories, the following technique is proposed. We represent in a normal probability plot the centred data (mean subtracted) of all sensors, so that those sensors with abnormal distance to the average appear displaced from the control line. As indicated in the methodology section, the first analysis is performed considering the average of all sensors (from #OUT to #11), while the second analysis considers only those sensors located inside the archaeological site (from #1 to #11). Notice that we do not analyse the distribution of all recorded data but only the differences from the average, which should be expected to follow a normal distribution.

**Figure 7.**
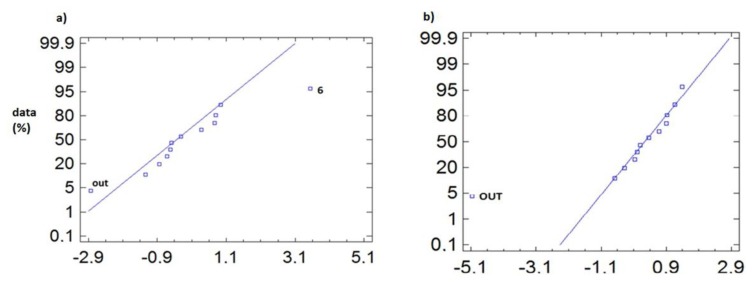
Normal probability plot of temperature, from February to July 2010, for centred data (subtracting the average from #OUT to #11). **(a)** From 8:00 to 19:59 h, with #6 and #OUT appearing as anomalous sensors (3,192 data/sensor); **(b)** from 20:00 to 7:59 h (3,192 data/sensor). At night, only #OUT stands out as anomalous sensor.

In Figure 7 we observe that for the temperature data, during the entire monitoring period, the outdoor sensor appears as abnormal, both for day and night. However, sensor #6 comes up as abnormal (Figure 7a) during daylight hours, as this sensor is immediately below the transparent cover, so that the incidence of sunlight causes an increase in temperature during the day.

Another option is to subtract the average obtained from all sensors inside the archaeological site, as they are the ones that will be taken as a reference, so we can avoid peculiarities introduced by the high variability and significant differences in mean of the outdoor sensor (#OUT). This approach allowed us to draw relevant conclusions, especially for the non-summer period (from February to May) where the temperature variability is lower and the inclusion of more extreme data, such as the outdoor sensor, in the calculation of the mean may bias the results and differences.

**Figure 8.**
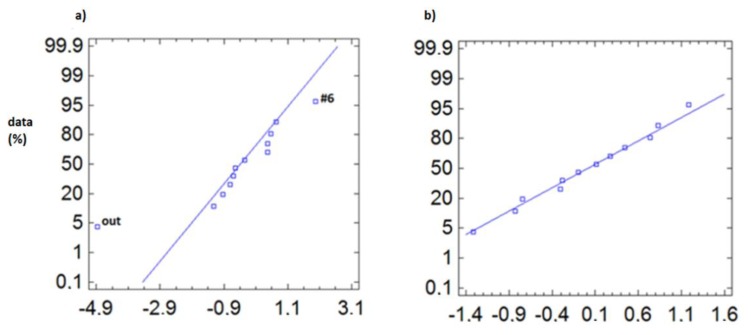
Normal probability plot of temperature for data from February to May 2010, from 8:00 to 19:59 (1,608 data/sensor), **(a)** data centred by the average of the inner sensors from (#1 to #11); **(b)** data centred by the average of the all sensors (from #OUT to #11).

Thus, in Figure 8, when considering the average of the inner sensors (Figure 8a), we can see how it is possible to appreciate the anomaly of sensors #6 and #OUT, whereas when the outdoor sensor is included in calculating the average (Figure 8b), these differences are concealed.

**Figure 9.**
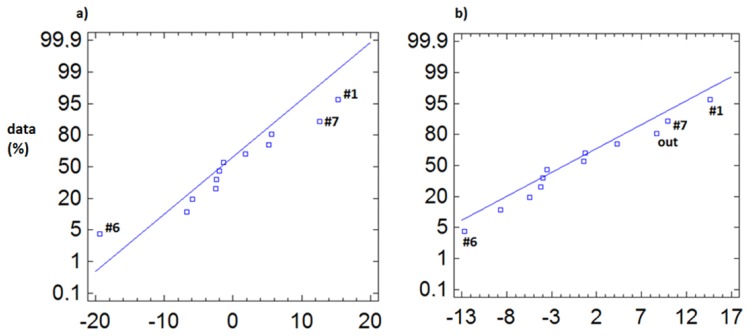
Normal probability plot of RH, for centred data (average of all sensors), from May to July, **(a)** from 8:00 to 19:59 h (1560 data/sensor); **(b)** from 20:00 to 7:59 h (1,560 data/sensor).

In the case of RH (Figure 9), the anomalous behaviour of sensor #6 is noteworthy, notably how the temperature increases due to the skylight, resulting in a drop in RH. On the other hand, sensors #1 and #7 present higher RH values, these sensors being located in the area closest to the water pipe.

No differences were found between the results of RH analyses for different statistics, because the RH average for outdoors was similar to the average for the inner sensors.

**Figure 10.**
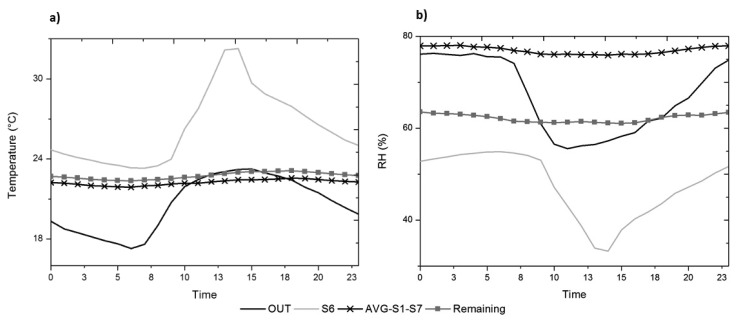
Mean daily trajectories, from May to July 2010. **(a)** For temperature data of sensors #OUT, #6 and the average of the remaining sensors; **(b)** for relative humidity (RH) data of sensors #OUT, the cluster composed by #1 and #7, sensor #6 and the average of the remaining sensors.

This technique for detecting singular trajectories, combined with the study of different factors (such as day/night cycles, seasons, or discussing the use of more accurate averages) allows us to specifically identify those sensors with different behaviour. However, to study and characterise the singular behaviour of these sensors in detail, below we analyse the mean daily trajectories (Figure 10).

It is notable how the trajectory shape of sensor #6 corresponds with that of the outdoor sensor, both in temperature and RH (Figure 10), accentuating the differences between night and day compared to all the other inner sensors, which have a highly damped trajectory. However, sensor #6 has a parallel offset with respect to the outdoor sensor, with an average temperature around 6 °C higher and a RH average about 20% lower, due to the overheating caused by the direct impact of sunlight, noticeably surpassing the values recommended by the standards [2,26].

On the other hand, the cluster formed by sensors #1 and #7 maintains a nearly constant value of RH throughout the day, but with values 18% higher than the rest of the inner sensors, due to the extra contribution of absolute humidity caused by the water pipe. This does not occur with temperature, which has very similar values to the other sensors.

#### 3.2.2. Analysis of the Remaining Sensors

Next, we carefully analyse possible differences, more subtle than those highlighted above, which may occur in the rest of the sensors inside the archaeological site. To do so, cluster analysis is performed on the average and mean daily variation of temperature and RH, so that is possible to empirically identify different clusters of sensors.

According to the study aim of identifying areas with singular behaviour inside the archaeological site, for temperature a solution with 4 clusters is selected. The solution was chosen after analysing the results for the highest and lowest number of clusters (Table 1) and the distance matrix (Table 2).

In Table 1 we can identify the sensors that comprise each cluster. As seen in Table 1, there are two clearly differentiated main clusters (C1, C4), whose distance from the centres of all clusters is always greater than 6 °C. C1 is the cluster that contains the outdoor sensor and is characterised by a high daily variability, while cluster C4 contains sensor #6, located under the skylight and characterised by very high temperature levels during daylight hours (which gives it a higher mean and a much higher variability than the rest of the archaeological site sensors).

**Figure 11.**
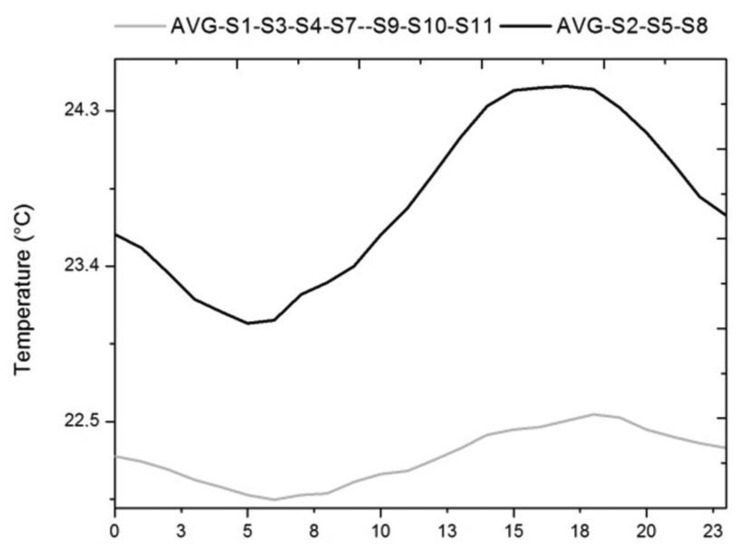
Mean daily trajectory of temperature for the average of sensors contained in cluster 2 and 3.

As seen in Table 1, there is a predominant cluster (C2) containing 7/12 of the sensors, and a second cluster (C3) of sensors similar to those of C2 (centre distance = 1.72 °C), but characterised by greater variability. Let us analyse the differences between these two clusters by comparing the mean daily trajectories (Figure 11).

Figure 11 shows the difference in averages previously indicated by cluster analysis. The difference in variability occurs due to an increase of temperature during daylight hours, caused by the proximity of the sensors to the skylight area. Thus, the trajectory shape of cluster 3 is similar to that of sensor #6 and the outdoor sensor, as shown in Figure 10. However, these sensors have an average approximately 3 °C lower than the average of sensor #6.

For RH, a solution with five clusters was selected (Table 3). The consistency of this solution can be determined by results shown in Table 4 (distance matrix). In Table 3 we can identify the sensors that comprise each cluster. As seen in Table 3, there are three different main clusters (C1, C2, C4), whose distance from the centres of all clusters is always greater than 10% of RH. C1 is the cluster that contains the outdoor sensor and is characterised by a high daily variability; cluster C2 is composed of sensors #1 and #7, those sensors located closer to the drain and which present the highest levels of RH (constant during all the day); finally, C4 contains sensor #6, located below the skylight and characterised by very low levels of RH during daylight hours (with a low average and high variability).

**Figure 12.**
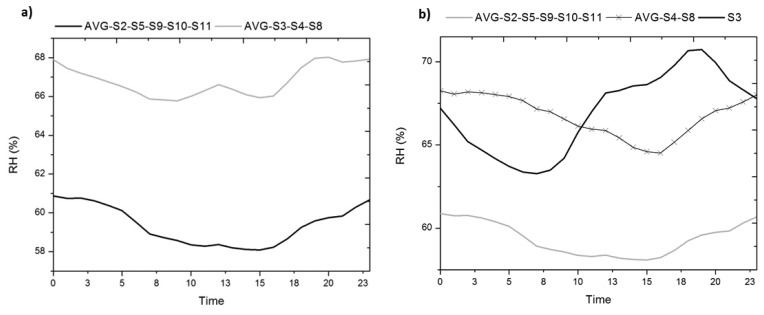
Mean daily trajectory of relative humidity of cluster analysis results. **(a)** The average of sensors contained in cluster 3 and 5; **(b)** The average of sensors contained in C3, and cluster 5, represented separately sensor #3 and average #4 and #8.

As seen in Table 3, there is a large cluster (C3) containing 5/12 of the sensors, and a second cluster (C5) of sensors similar to cluster C3 (centre distance = 7.58% of RH), but characterised by a higher average and variability. Let us analyse the differences between these two clusters comparing the mean daily trajectories (Figure 12).

**Figure 13.**
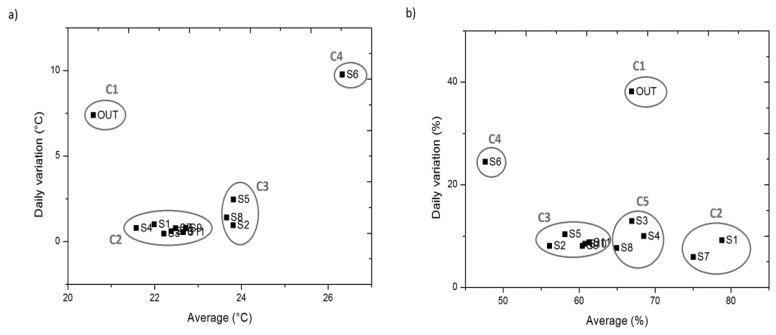
Bivariate plot of average *versus* mean daily variation, from 22 February 2010 to 5 May 2010, from 00:00 to 23:59 h (6,384 data points/sensor), **(a)** temperature; **(b)** relative humidity.

An alternative approach for identifying clusters of sensors is proposed. Next, we represent the bivariate plots of temperature and RH for the average *versus* the mean daily variation. Thus, we can identify visually in Figure 13 different clusters of sensors, coinciding with the results obtained by cluster analysis.

As shown in Figure 12, cluster 5 is composed by sensors #3, #4 and #8 due mainly to having an average greater than C3. By means of the bivariate plots (Figure 13) we observe that sensor #3 has more variability, a parameter that captures the amplitude of the trajectory but not its form. Thanks to the representation of the mean daily trajectories (Figure 12b), we can see the singular form of the trajectory of sensor #3 which, contrary to what happens to the rest of the sensors, undergoes an increase of approximately 8% of RH during the day. This occurs to sensor #3 because it is in the direct path of the air outlets of the climate control system, so its trajectory reflects the system shutdowns and the cooling strategies followed.

This approach enables us to identify clusters of sensors with similar average and amplitude, as well as allowing comparisons between RH and temperature. Here, it is important to mention the difference between #5 and #8 in temperature, where these sensors are strongly influenced by the entry of light from the skylight. However, the results in RH differ from those obtained for temperature, as the water pipe apparently softens the effect of the temperature rise caused by the skylight (which should result in lower RH values).

## 4. Conclusions

Characterising an archaeological site is of great importance, with the aim, on one hand, of carrying out comparative studies in the future when implementing changes in climate control systems or in the architectural design, and on the other hand, to study any future deterioration of the archaeological site, which may be related with microclimate conditions.

The complexity of the data collected and the limitation of the location of the sensors make it difficult to draw relevant and reliable conclusions through standard techniques, such as contour plots or temporal trajectory analysis. The present work is mainly intended for restorers and conservators of archaeological sites.

Three statistical methodologies that are simple to operate have been proposed (two qualitative and one quantitative), which can replace more complex multivariate statistical techniques such as cluster analysis. These proposed techniques are normal probability plot (quantitative), bivariate plots and mean daily trajectories (qualitative), which have been useful in characterising the archaeological site in detail, highlighting the differences between areas.

The results of these techniques have revealed the significant influence of the skylight on the temperature and RH, causing sharp rises and falls during daylight hours. Sensors placed in the vicinity of the cover, but not immediately below, have different behaviour from the other inner sensors. In the case of sensor #3, it was possible to detect the direct impact of air from the conditioning system and how the trajectory reflects its operation. Possible solutions to this problem might be installing an external cover over the skylight, painting the glass of the skylight, *etc.*

On the other hand, a boundary water pipe clearly configures an RH gradient, which decreases as we move away from the pipe. This effect is important to emphasise, as the presence of old water pipes in urban archaeological sites should not be unusual. Piping makes regular monitoring necessary because water may leak, affecting the conservation of the archaeological sites.

When implementing any solution, it would be advisable to perform a short-term monitoring during summer (since it is the most conflictive microclimatic period) and especially in the area immediately below the skylight and its surroundings. Since the current number of sensors has allow us reaching useful conclusions we consider this an adequate proportion of sensors (in terms of cost-result) whenever working with the appropriate techniques.

## Figures and Tables

**Table 1.** Results of cluster analysis of temperature for data from May to July, from 00.00 to 23.59 h (3,120 data points/sensor).

**Cluster**		**C1**	**C2**	**C3**	**C4**
Sensors		OUT	1,3,4,7,9,10,11	2,5,8	6
Centres (°C)	average	20.59	22.30	23.76	26.33
	Daily variation	7.39	0.71	1.61	9.76
Fraction of sensors		1/12	7/12	3/12	1/12

**Table 2.** Matrix of distances (in °C) between the final cluster centres for cluster analysis of temperature data.

	**C2**	**C3**	**C4**
C1	6.89	6.60	6.21
C2		1.72	9.91
C3			8.55

**Table 3.** Results of cluster analysis of RH for data from May to July 2010, from 00.00 to 23.59 h (3,120 data points/sensor).

**Cluster**		**C1**	**C2**	**C3**	**C4**	**C5**
Sensor		OUT	1,7	2,5,9–11	6	3,4,8
Centres (%)	average	66.93	76.94	59.37	47.64	66.82
	daily variation	38.13	7.54	8.73	24.43	10.18
Fraction of sensors		1/12	2/12	5/12	1/12	3/12

**Table 4.** Matrix of distances (in % of relative humidity (RH)) between the final cluster centres for cluster analysis of RH data.

	**C2**	**C3**	**C4**	**C5**
C1	32.18	30.35	23.66	27.95
C2		17.61	33.82	10.46
C3			19.60	7.58
C4				23.89
